# Anion‐Dependent Polarization and Piezoelectric Power Generation in Hybrid Halide MAPbX_3_ (X = I, Br, and Cl) Thin Films with Out‐of‐Plane Structural Adjustments

**DOI:** 10.1002/advs.202204462

**Published:** 2022-12-01

**Authors:** Da Bin Kim, Kyeong Su Jo, Kwan Sik Park, Yong Soo Cho

**Affiliations:** ^1^ Department of Materials Science and Engineering Yonsei University Seoul 03722 Republic of Korea; ^2^ Department of Electrical and Computer Engineering University of Toronto Toronto Ontario M5S 3G4 Canada

**Keywords:** energy harvester, MAPbI_3_, perovskite halide, piezoelectricity, strain engineering

## Abstract

Anion‐dependent differences in the electromechanical energy harvesting capability of perovskite halides have not been experimentally demonstrated thus far. Herein, anion‐dependent piezoelectricity and bending‐driven power generation in high‐quality methylammonium lead halide MAPbX_3_ (X = I, Br, and Cl) thin films are explored; additionally, anisotropic in situ strain is imposed to improve energy harvesting under tensile bending. After applying the maximum in situ strain of −0.73% for all the halide thin films, the MAPbI_3_ thin‐film harvester exhibited a peak voltage/current of ≈23.1 V/≈1703 nA as the best values, whereas MAPbBr_3_ and MAPbCl_3_ demonstrated ≈5.6 V/≈176 nA and ≈3.3 V/≈141 nA, respectively, under identical bending conditions. Apart from apparent ferroelectricity of tetragonal MAPbI_3_, origin of the piezoelectricity in both cubic MAPbBr_3_ and MAPbCl_3_ is explored as being related to organic–inorganic hydrogen bonding, lattice distortion, and ionic migration, with experimental supports of effective piezoelectric coefficient and grain boundary potential. Conclusively, piezoelectricity of the cubic halides is assumed to be due to their soft polarity modes and relatively low elastic modulus with vacancies contributing to space‐charge polarization. In the case of ferroelectric MAPbI_3_, the distortion of PbI_6_ octahedra and atomic displacement within each octahedron are quantitatively estimated.

## Graphical abstract

Experimental evidence and origin of the anion‐dependent piezoelectricity in methylammonium lead haldie MAPbX_3_ (X = I, Br, and Cl) thin films are proposed for the first time. Their electromechanical power‐generating capability is significantly enhanced by applying both vertical tensile lattice strain and an optimal poling field, with the noticeable best values of 23.1 V and 1703 nA.

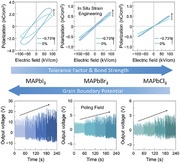

## 1 Introduction

Thin films based on halide perovskite materials (ABX_3_ where A = CH_3_NH_3_, (NH_2_)_2_CH, or Cs; B = Pb, Sn, or Ge; and X = Cl, Br, or I) have been actively investigated as active layers for flexible applications in solid‐state lighting,^[^ ^1^, ^2^ ^]^ solar energy conversion,^[^ ^3^, ^4^ ^]^ photodetectors,^[^ ^5^, ^6^, ^7^ ^]^ and electromechanical energy conversion.^[^ ^8^ ^]^ Such halides can be processed at low temperatures, which makes them compatible with various plastic substrates, such as polyethylene terephthalate (PET), polyethylene naphthalate (PEN), and polyimide (PI). Thus, for piezoelectric and related devices, these materials have great potential for flexible systems that cannot easily be assembled from typical oxide‐based piezoelectric materials.^[^ ^9^, ^10^, ^11^, ^12^ ^]^ Particularly, piezoelectric energy harvesters composed of flexible halide thin films on plastic substrates have been recently reported for a variety of halide compounds, such as CsPbBr_3_,^[^ ^13^ ^]^ CsSnI_3_,^[^ ^14^ ^]^ and MASnI_3_.^[^ ^15^ ^]^ However, a wider range of halide compounds is not yet available, presumably because of the difficulty in obtaining high‐quality thin films from limited chemical precursors and synthesis conditions. Owing to the flexibility of halide harvesters, they enable the use of mechanical bending or pressing as the source of the input force for electromechanical coupling, whereas mechanical vibrations are usually the source for typical piezoelectric oxide films on rigid substrates such as Si.

The bending‐driven power generation of halide thin films has been quite impressive. For example, CsPbBr_3_ thin films with a thickness of ≈260 nm delivered 16.4 V and 604 nA as a result of a bending strain of 1.67% at a frequency of 1 Hz.^[^ ^13^ ^]^ In the case of ≈354‐nm‐thick CsSnI_3_ thin films, manually bending the harvesters achieved output values of 9.5 V and 445 nA.^[^ ^14^ ^]^ However, the research on perovskite halide thin films, particularly concerning piezoelectricity and related power generation, is in its early stages, although its high potential has been recognized. There have been a few examples describing the effects of halide constituents on piezoelectricity of organic–inorganic hybrid halides but it is hard to find the clear dependency of such compositional variations on the electromechanical energy harvesting performance.^[^ ^16^, ^17^ ^]^ For example, the partial substitution of I with Cl in (FMTMA)PbI_3_, where FMTMA is (ferrocenylmethyl)trimethylammonium, resulted in more octahedral distortion and higher piezoelectricity, which led to a noticeable output voltage of ≈4 V.^[^ ^16^ ^]^

Herein, we explore the anion‐dependent piezoelectric properties of MAPbX_3_ (X = I, Br, and Cl) thin films for the first time, and we associate these properties with their electromechanical energy harvesting characteristics. Recently, the effects of these three different anions on the structural and optical characteristics of MAPbX_3_ halides, such as their lattice parameters, ion migration, energy bandgap, and optical absorption, have been examined, but mostly based on theoretical estimations.^[^ ^18^, ^19^, ^20^, ^21^, ^22^ ^]^ The piezoelectricity and related device performance of the halide compounds of MAPbBr_3_ and MAPbCl_3_, however, have not been investigated, although an output voltage of 7.29 V was reported for a 500‐nm‐thick MAPbI_3_ thin film.^[^ ^23^ ^]^ Therefore, it would be very interesting to compare the polarization behavior of the anion‐variable halide compositions and their effects on piezoelectricity, particularly with regard to power generation.

In addition to determining the origin of piezoelectricity in MAPbX_3_ halides, two technical approaches have been adopted to enhance their electromechanical energy harvesting performance: the in situ strain engineering to introduce vertical tensile strain and the application of electric field for extra polarization. The out‐of‐plane structural adjustments are expected to be useful in extending octahedral networks in perovskites as domain formation proceeds in a tensile‐stress atmosphere (created by bending for energy harvesting) when sandwiched electrodes are applied. In situ strain was recently imposed using a curved plastic substrate during the two‐step synthesis of inorganic halide CsPbBr_3_ films; this proposed technique was promising for increasing piezoelectricity when a maximum anisotropic strain of 0.75%, that is, in‐plane compressive strain and out‐of‐plane tensile strain, was introduced into ≈545‐nm‐thick film.^[^ ^24^ ^]^ With applying the optimal in situ strain and electric field through this work, MAPbI_3_ thin films demonstrate the highest peak output values of ≈23.1 V and ≈1703 nA, in contrast with the low values of 5.6 V and 176 nA for MAPbBr_3_ and 3.3 V and 141 nA for MAPbCl_3_. The output values for MAPbI_3_ are the best values reported for any halide thin‐film‐based energy harvesters. The origin of the observed piezoelectricity in all three halides is explored in conjunction with lattice distortion, polarization, and grain boundary potentials, and in particular, the distortion of PbI_6_ octahedra in the case of tetragonal MAPbI_3_ is further correlated with its performance.

## 2 Results and Discussion

**Figure 1:**
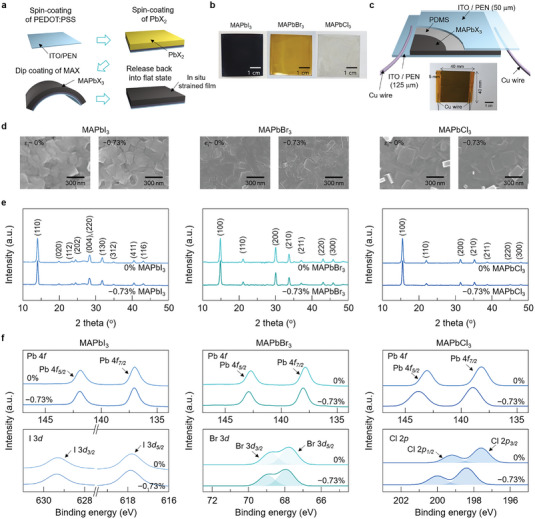
a) Schematic of the two‐step deposition process imposing in situ lattice strain in the synthesized MAPbX_3_ (X = I, Br, and Cl) thin films, wherein PbX_2_ is first deposited on a flat PEDOT:PSS/ITO/PEN substrate, and MAX is subsequently coated onto the bent PbX_2_ layer (in this example compressive strain is created in the in‐plane direction by depositing MAX onto a convexly bent film). In the final step, the bent film is released back into the flat state. b) Actual photographs of dark gray MAPbI_3_, yellow MAPbBr_3_, and gray MAPbCl_3_ films. c) Schematic of a piezoelectric energy harvester with the structure of PEN/ITO/PDMS/MAPbX_3_/PEDOT:PSS/ITO/PEN. d) Surface SEM images of each type of halide thin film processed with 0% strain (left) and an in situ strain *ε_i_* of −0.73% (right). e) XRD patterns of the unstrained and −0.73%‐strained films. f) Selected XPS spectra of the Pb 4*f* and I 3*d* states in MAPbI_3_, the Pb 4*f* and Br 3*d* states in MAPbBr_3_, and the Pb 4*f* and Cl 2*p* states in MAPbCl_3_ for the unstrained and −0.73%‐strained cases.

A two‐step deposition method, which consisted of first spin‐coating PbX_2_ and then dip‐coating MAX, was used to synthesize flexible MAPbX_3_ (X = I, Br, and Cl) thin films on an indium tin oxide (ITO)‐coated PEN substrate. In addition, to impose in situ strain, the PbX_2_ film was bent between the two deposition steps. **Figure** **1**a illustrates the critical steps for preparing the in situ strained MAPbX_3_. Specifically, the PbX_2_ layer was first spin coated onto poly(3,4‐ethylenedioxythiophene) polystyrene sulfonate (PEDOT:PSS)/ITO/PEN. The assembly was then bent using a fixture with a given curvature and inserted into the MAX solution for the final synthesis of MAPbX_3_ thin films. After drying at 50 °C, the bent films were released back into a flat state. Photographs of the resultant films of dark gray MAPbI_3_, yellow MAPbBr_3_, and gray MAPbCl_3_ are shown in Figure 1b, all of which exhibit rather uniform coverage over an area of a few centimeters. After depositing polydimethylsiloxane (PDMS) on the synthesized halide films, the final harvester structure of PEN/ITO/PDMS/MAPbX_3_/PEDOT:PSS/ITO/PEN was completed with another top ITO/PEN substrate, as schematically presented in Figure 1c, along with a photograph of an actual harvester with an effective area of 3.5 cm × 4 cm.

The type of in situ strain *ε_i_* can be controlled by the direction of the bent substrate (concavely vs convexly), while the level of curvature (a larger strain with a smaller bent curvature) determines the magnitude. The type and magnitude of *ε_i_* was initially optimized using MAPbI_3_ in the *ε_i_* range of +0.73% to −0.73%. As the optimal strain, a maximum compressive *ε_i_* of −0.73% was attained by convexly bending the substrate with a curvature of 8.2 mm, where the negative sign of *ε_i_* implies compressive strain in the lateral direction. Similarly, the maximum compressive strain in the lateral direction was previously reported to be optimal for vertical lattice extension and thus the best out‐of‐plane piezoelectricity.^[^ ^25^ ^]^ Accordingly, a maximum strain of −0.73% was applied to the other halides, MAPbBr_3_, and MAPbCl_3_. Figure 1d shows the scanning electron microscope (SEM) images of the surface of halide thin films processed with in situ *ε_i_* values of 0% and −0.73%. Distinct grain structures with few voids are evident in all the halide thin films, regardless of the in situ strain. Cross‐sectional SEM images of the MAPbX_3_ films are presented in Figure S1 Supporting Information, confirming the good adhesion of the densely packed films to the PEDOT:PSS/ITO/PEN substrate. The film thicknesses were ≈486, ≈464, and ≈450 nm for MAPbI_3_, MAPbBr_3_, and MAPbCl_3_, respectively.

Figure 1e shows the X‐ray diffraction (XRD) patterns of halide thin films processed with *ε_i_* = 0% and −0.73%. All films were phase‐pure perovskite structures: tetragonal *I4/mcm* MAPbI_3_, cubic *Pm* $\bar{3}$ *m* MAPbBr_3_, and cubic *Pm* $\bar{3}$ *m* MAPbCl_3_.^[^ ^26^ ^]^ A polycrystalline nature with no preferred orientation was clearly observed, with strong peaks for the (110) plane of MAPbI_3_ and for the (100) planes of MAPbBr_3_ and MAPbCl_3_. Although applying a compressive strain of −0.73% did not affect the development of the perovskite phase, this extra compressive strain changed the lattice parameters, thus shifting the XRD peaks. For example, Figure S2, Supporting Information shows highlighted high‐resolution (HR)‐XRD peaks for the (004) and (220) planes of tetragonal MAPbI_3_, wherein the (004) peak clearly shifted to lower 2*θ* angles with *ε_i_* = −0.73%, whereas the (220) peak moved in the opposite direction. The XRD patterns of MAPbBr_3_ and MAPbCl_3_ also exhibit the anticipated shifts in the peaks for the (100) plane at *ε_i_* = −0.73%. Further XRD patterns and photoluminescence (PL) spectra only for strained MAPbI_3_ are available in Figure S3, Supporting Information, verifying that the peaks gradually shifted with the in situ strain over the extended tensile‐to‐compressive strain range of +0.73% to −0.73%.

The existence of in situ strain in the halide thin films was confirmed by X‐ray photoelectron spectroscopy (XPS). Selected XPS spectra of the Pb 4*f* and I 3*d* states are shown in Figure 1f for the MAPbI_3_ thin films processed with *ε_i_* of 0% and −0.73%, in which the Pb 4*f*, I 3*d*_3/2_, and I 3*d*_5/2_ peaks all shifted to higher binding energies with a compressive strain of −0.73%. Identical shifts were observed in the XPS spectra of the −0.73%‐strained MAPbBr_3_ and MAPbCl_3_ thin films. Indeed, all the peaks of the Pb 4*f* states (4*f*_5/2_ and 4*f*_7/2_), Br 3*d* states (3*d*_3/2_ and 3*d*_5/2_), and Cl 2*p* states (2*p*_1/2_ and 2*p*_3/2_) shifted toward higher binding energies with the application of *ε_i_*, which indicates that the lattice contraction incurred a stronger binding energy in all halide thin films.

**Figure 2:**
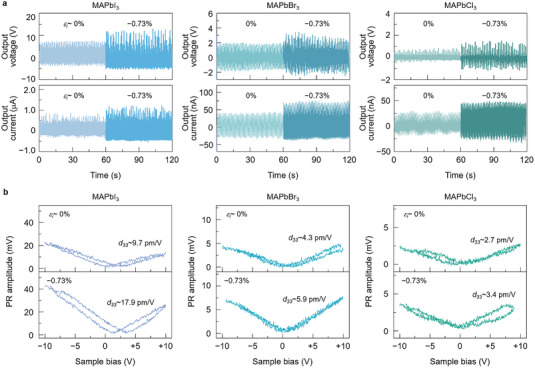
a) Output voltage and current generated for the unstrained, and−0.73%‐strained MAPbI_3_, MAPbBr_3_, and MAPbCl_3_ thin films, which were measured during bending at a bending strain and frequency of 0.47% and 2.9 Hz, respectively. b) PR amplitude–bias behavior of the unstrained and −0.73%‐strained MAPbI_3_, MAPbBr_3_, and MAPbCl_3_ thin films as characterized by PFM, with the calculated effective piezoelectric coefficient *d*_33,eff_ from each peak PR value.

Electromechanical energy harvesters based on in situ strained MAPbX_3_ thin films were fabricated to investigate the influence of *ε_i_*, on the energy harvesting characteristics. The harvesting performance produced by bending the harvesters with variable curvatures largely depended on the type and magnitude of *ε_i_*, as shown in the plots of the output voltage and current values in **Figure** **2**a. The best results were achieved by introducing an in situ compressive strain of −0.73%, which substantially increased the voltage and current, with the −0.73%‐strained MAPbI_3_ attaining the highest values of ≈12.9 V and ≈1283 nA, respectively, under the optimal bending conditions, that is, a bending frequency and strain of 2.9 Hz and 0.47%, respectively. These values correspond to increments of ≈74% and ≈64%, respectively, relative to the values of ≈7.4 V and ≈780 nA for the unstrained harvester. As an opposite case from the compressive strain, the tensile in situ strain of +0.73% resulted in the worst energy harvesting performance in all the halide films as seen in Figure S4, Supporting Information, where a vertical lattice contraction is expected. Previously reported output voltage and current values for MAPbI_3_‐based thin‐film harvesters ranged from 0.5 to 7.29 V and from 50 to 880 nA, respectively,^[^ ^23^, ^27^, ^28^, ^29^ ^]^ which indicates that our values of ≈7.4 V and ≈780 nA for the unstrained sample belong in the reported ranges. The long‐term harvesting performance with variations in the type and magnitude of *ε_i_*, bending frequency, and bending strain is presented in Figures S5–S7, Supporting Information for the MAPbI_3_ thin films. With bending operations of up to 20 000 cycles, the output voltage was maintained well, as shown in Figure S8, Supporting Information. The polarity switching behavior of the harvester shown in Figure S9, Supporting Information suggests that the harvesting outcomes were produced by the halide films.

Compared to that of the MAPbI_3_ thin films, the harvesting performance of the MAPbBr_3_ and MAPbCl_3_ thin films was far inferior, although the introduction of *ε_i_* substantially improved the power‐generation capability. The peak voltage/current values were ≈1.9 V/≈44 nA for MAPbBr_3_ and ≈0.8 V/≈32 nA for MAPbCl_3_, but the compressive strain of −0.73% increased these values to ≈3.5 V/≈76 nA for MAPbBr_3_ and ≈1.4 V/≈50 nA for MAPbCl_3_. The piezoelectric energy harvesting performance of MAPbBr_3_ and MAPbCl_3_ thin films has not been reported thus far. Therefore, it is interesting that MAPbBr_3_ and MAPbCl_3_ exhibited piezoelectric power generation outcomes despite of cubic *Pm* $\bar{3}$ *m* perovskite structure. A few studies have been conducted on the piezoelectricity of organic–inorganic cubic halide perovskites, namely, MA‐ or formamidinium (FA)‐based compositions, as summarized with reported values of piezoelectric coefficient *d*_33_ in Table S1, Supporting Information.^[^ ^30^, ^31^, ^32^ ^]^ Concerning piezoelectricity, the unusual hydrogen bonds between the MA^+^ (or FA^+^) and X^−^ ions, which induce local asymmetry, are regarded to be responsible for the presence of piezoelectricity even in cubic perovskites. Specifically, the soft phonon (polar) modes with smaller moduli from the organic ions provide high flexibility, thus enabling lattice distortion (i.e., ionic displacement and rotation) under a mechanical stimulus for induced dipole moments.^[^ ^33^, ^34^ ^]^ MA^+^ cations are known to possess a dipole moment of 2.29 Debye ( = 7.64 × 10^−30^ C m) owing to the uncompensated local charge distribution in MABX_3_ perovskites.^[^ ^35^ ^]^ The piezoelectricity in the hybrid halides is also associated with the space‐charge polarization concerning ionic defects as will be discussed later. As reference, we measured dielectric constant *ε_r_* of the three halide films with the variation in frequency as presented in Figure S10, Supporting Information. The *ε_r_* values at 1 kHz were ≈67 for MAPbI_3_, ≈43 for MAPbBr_3_, and ≈27 for MAPbCl_3_, which indicates the higher dipole moment in MAPbI_3_ than the other halides.

To confirm the piezoelectricity of the cubic MAPbBr_3_ and MAPbCl_3_ thin films, we characterized these samples using piezoresponse force microscopy (PFM), as shown in the piezoresponse (PR) amplitude versus DC bias (±10 V) plots in Figure 2b. The resultant PR amplitude and phase images over a scanning area of ≈3 µm × 3 µm are shown in Figures S11 and S12, Supporting Information. The image of the film with larger compressive *ε_i_* demonstrated brighter contrast, reflecting the higher PR amplitudes, whereas the images became progressively darker toward the tensile *ε_i_* region.^[^ ^36^ ^]^ Figure 2b shows the nonlinear behavior of amplitudes with the applied DC bias varying from −10 V to +10 V for the strained MAPbI_3_, MAPbBr_3_, and MAPbCl_3_ thin films, as measured by PFM. Stronger nonlinear behavior with larger amplitudes is evident over the DC bias range with the compressive *ε_i_*. The effective piezoelectric coefficient *d*_33,eff_ was estimated using the peak amplitude value of each strained film, and *d*_33,eff_ increased with the compressive *ε_i_* of −0.73% relative to the that of the corresponding unstrained halide film, specifically, from ≈9.7 to ≈17.9 pm V^−1^ for MAPbI_3_, ≈4.3 to ≈5.9 pm V^−1^ for MAPbBr_3_, and ≈2.7 to ≈3.4 pm V^−1^ for MAPbCl_3_. A similar *d*_33,eff_ value of 6 pm V^−1^ was previously observed with MAPbI_3_ thin films using PFM.^[^ ^37^ ^]^ In addition, *d*_33_ values of 31.4 pC N^−1^ for MAPbI_3_ and 6.7 pC N^−1^ for MAPbCl_3_ have been reported on the basis of theoretical calculations.^[^ ^38^ ^]^ The low levels of energy harvesting in MAPbBr_3_ and MAPbCl_3_ thin films are likely related to their low piezoelectric coefficients. Note that the PFM measurements reflect the vertical piezoelectric response, so the vertical lattice extension with compressive *ε_i_* helps induce larger piezoelectricity.

**Figure 3:**
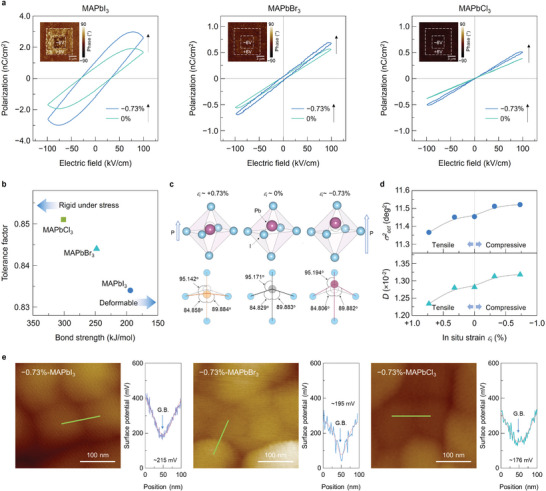
a) Polarization–electric field curves of the −0.73%‐strained MAPbI_3_, MAPbBr_3_, and MAPbCl_3_ thin films, which demonstrate apparent ferroelectric behavior only in MAPbI_3_, with the inserted PFM phase images characterized by applying consecutive biases of +6 and −6 V for the designated box areas. b) Plot of tolerance factor versus bond strength for MAPbI_3_, MAPbBr_3_, and MAPbCl_3_. c) Schematics of the PbI_6_ octahedron in the case of MAPbI_3_ with *ε_i_* = +0.73% (tensile), 0%, and −0.73% (compressive) strain, illustrating the *ε_i_*‐dependent position of the off‐centered Pb ion and concomitant changes in the I—Pb—I (an out‐of‐plane bond) and I—Pb—I (two planar bonds) bond angles. d) *ε_i_*‐dependent changes in the bond angle variance *σ^2^*_oct_ (deviation from the normal bond angles within the octahedron) and distortion index *D* (deviation from the normal average length of all six Pb—I bonds in the octahedron) for MAPbI_3_. All estimated values are available in Table S2, Supporting Information. e) Changes in the surface potential across the grain boundary in the −0.73%‐strained halide films, as characterized by KPFM.

We also investigated the polarization behavior of the halide thin films, as shown in the polarization–electric field (P–E) curves in **Figure** **3**a. As expected, clear ferroelectricity with hysteresis behavior was evident in MAPbI_3_, whereas no ferroelectricity was apparent in MAPbBr_3_ and MAPbCl_3_.^[^ ^23^, ^39^ ^]^ The current–bias curves confirm the nonlinear or linear dependence of the polarization behavior, as shown in Figure S13, Supporting Information.^[^ ^40^ ^]^ The apparent ferroelectricity of MAPbI_3_ thin film was also confirmed by applying consecutive biases of +6 and −6 V for the designated box areas as seen in the inserted PR phase images of Figure 3a, where only the case of MAPbI_3_ demonstrated obvious contrast changes with switching the bias direction. The anion‐dependent polarization may also be associated with the tolerance factor and the Pb—X bond strength, both which increase in the order of I < Br < Cl, as shown in Figure 3b. The higher tolerance factor with Cl comes from the smaller ionic radius of Cl^−^ ions relative to that of the other ions in the perovskite structure (namely 181 pm for Cl^−^, 196 pm for Br^−^, and 220 pm for I^−^).^[^ ^41^ ^]^ With a higher Pb—Cl bond strength, the [PbX_6_]^4−^ frameworks in MAPbCl_3_ are expected to be less deformable than those in the other halides, thus suggesting the lowest piezoelectric effect.^[^ ^42^, ^43^ ^]^ Because the central cavity occupied by the MA^+^ cation in the perovskite structure is small, and because the large electronegativity of Cl induces strong hydrogen bonds of N—H⋯X, MA^+^ may undergo limited deformation in MAPbCl_3_ under external stress. It should be also mentioned that the reported Young's moduli, that is, 15.9 GPa for MAPbI_3_, 17.8 GPa for MAPbBr_3_, and 19.9 GPa for MAPbCl_3_, are correlative to the changing trend concerning deformable nature in the order of I > Br > Cl.^[^ ^44^, ^45^ ^]^ Conversely, MAPbI_3_ is assumed to exhibit the best piezoelectric behavior owing to its higher structural adaptability, which allows more lattice distortion under an external electrical or mechanical source.

Note that the larger piezoelectricity in tetragonal *I4/mcm* MAPbI_3_ was exclusively due to the existence of permanent dipoles and the increase in dipole moments with the applied vertical tensile strain, as discussed above. Therefore, we could extend our experiment only to MAPbI_3_ to determine the distortion of permanent dipoles (or PbI_6_ octahedra). Based on the *ε_i_*‐dependent crystallographic parameters obtained by XRD analysis (Table S2, Supporting Information), structural information such as bond angles and octahedral distortion in tetragonal MAPbI_3_ was obtained using Visualization for Electronic and Structural Analysis (VESTA) software.^[^ ^46^ ^]^ Table S3, Supporting Information lists the calculated structural parameters, such as the I—Pb—I bond angles in the equatorial (in‐plane) and vertical (out‐of‐plane) directions and Pb—I bond lengths within a PbI_6_ octahedron. Figure 3c schematically illustrates the structural changes in the PbI_6_ octahedron for the *ε_i_* cases of +0.73%, 0%, and −0.73%, labeled with the average values of the appropriate bond angles. For example, compressive *ε_i_* reduced the in‐plane I—Pb—I angles and increased the out‐of‐plane I—Pb—I angle within the octahedron, indicating the vertical extension of the octahedron. The vertical extension with compressive *ε_i_* is likely to accompany a larger transverse displacement of Pb ions from the center of the octahedron, which must be associated with enhanced piezoelectricity. Further, two other parameters, that is, the bond angle variance *σ^2^*_oct_ (indicating the deviation in the normal angle between Pb and I) and the distortion index *D* (indicating the deviation from the average length of all six Pb—I bonds within an octahedron), were extracted using the following relations.^[^ ^47^, ^48^ ^]^

(1) $$\sigma_{\mathrm{oct}}^{2}=\frac{1}{11}\;\sum_{i\;=\;1}^{12}{\left(\alpha_{i}-90\right)}^{2}$$

(2) $$D\;=\frac{1}{6}\;\sum_{i\;=\;1}^{6}\frac{|l_{i}-l_{\mathrm{av}}|}{l_{\mathrm{av}}}$$

where *α*_i_ is the individual I—Pb—I angle, *l*_av_ is the average length of Pb—I bonds, and *l*_i_ is the individual bond length. Figure 3d shows the variations in *σ^2^*_oct_ and *D* as a function of *ε_i_*. As expected, the case with −0.73% exhibited values of *σ^2^*_oct_ ≈ 11.52 deg^2^ and *D* ≈ 1.32 × 10^−2^, both of which were higher than those of the unstrained reference, 11.45 deg^2^ and 1.28 × 10^−2^. These higher values suggest more distortion of individual octahedra with a higher compressive *ε_i_*, with extensive off‐centering of the central Pb atoms in the octahedral.^[^ ^13^, ^49^, ^50^ ^]^

Further, the surface potential distributions across the grain boundary were characterized by Kelvin probe force microscopy (KPFM) for the −0.73%‐strained MAPbX_3_ thin films, as shown in the line profiles of the surface potential in Figure 3e. A lower surface potential was observed at the grain boundaries of the halides, implying an even distribution of surface charges across the grain boundary, as similarly reported for perovskite halides.^[^ ^51^, ^52^, ^53^ ^]^ This lowered potential may be attributed to the extensive formation of ionic defects in the grain regions, which can accumulate near the grain boundary. Perovskite halides are known to easily form halogen vacancies such as V'_I_, V'_Br_, and Vʹ_Cl_, leading to n‐type semiconductor behavior.^[^ ^54^, ^55^, ^56^, ^57^ ^]^ The surface potential differences in the halides may indicate the contributions of defects, with a higher potential owing to the higher concentration of defects. The surface potentials of ≈215 mV for MAPbI_3_, ≈195 mV for MAPbBr_3_, and ≈176 mV for MAPbCl_3_ indicate that defect formation may be more extensive in MAPbI_3_, as suggested by the lowest bond strength of Pb—I compared to the corresponding bonds in the other halides.^[^ ^20^, ^58^ ^]^ We therefore measured the carrier mobility of the halides to ascertain the contribution of defects to the potential differences. As expected, MAPbI_3_ showed a higher carrier mobility of 51.4 cm^2^ V^−1^ s^−1^, in contrast with 36.7 cm^2^ V^−1^ s^−1^ for MAPbBr_3_ and 13.9 cm^2^ V^−1^ s^−1^ for MAPbCl_3_, as a result of Hall measurements. Such defects may contribute ultimately to the formation of space‐charge polarization by ionic migrations under an electric field.

**Figure 4:**
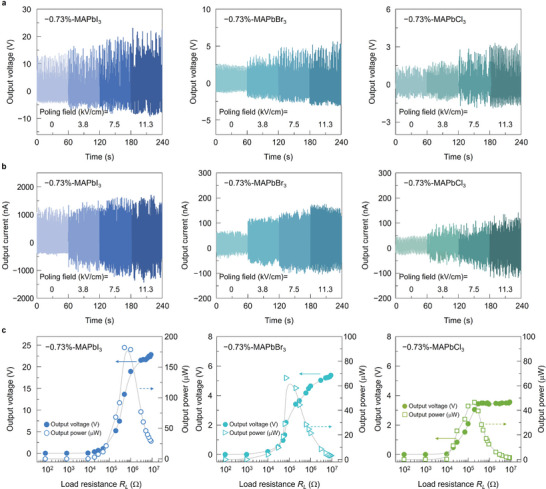
a) Output voltage and b) output current generated while applying poling fields of 0–11.3 kV cm^−1^ for the −0.73%‐strained MAPbI_3_, MAPbBr_3_, and MAPbCl_3_ thin films, which were measured during periodic bending at a strain and frequency of 0.47% and 2.9 Hz, respectively. c) Output voltage and power measured with the load resistance varying from 10^2^ to 10^7^ Ω for the −0.73%‐strained MAPbI_3_, MAPbBr_3_, and MAPbCl_3_ thin films.

Finally, we applied electric field to align dipoles vertically in the halide films to maximize piezoelectric energy harvesting. **Figure** **4**a,b shows the variations in the output voltage and current of the −0.73%‐strained halide films with an increasing electric field of up to 11.3 kV cm^−1^. As expected, the output values tended to increase with the increasing magnitude of the electric field, reaching maximum values of 23.1 V/1703 nA for MAPbI_3_, 5.6 V/176 nA for MAPbBr_3_, and 3.3 V/141 nA for MAPbCl_3_ at the maximum field of 11.3 kV cm^−1^. The applied electric field is believed to act as a poling field for domain alignments only in ferroelectric MAPbI_3_. However, the field‐driven harvesting enhancements in non‐ferroelectric MAPbBr_3_ and MAPbCl_3_ are likely understood as being associated with the further distortion of local asymmetry structure and the redistribution of defects under an electric field as reported in organic–inorganic cubic perovskite halides.^[^ ^59^, ^60^, ^61^ ^]^ For example, the enhanced positive polarization was observed by ion redistribution under an applied electric field in cubic MAPbBr_3_ crystals, where the cations move along the direction of the applied electrical field while the anions travel to the opposite direction, resulting in the accumulated charges on one side.^[^ ^59^ ^]^ There is another report dealing with the effect of electric field‐driven ion migration in cubic MAPbCl_3_ single crystals.^[^ ^60^ ^]^ In the case of cubic MAPb(I_0.88_Br_0.12_)_3_ single crystal, electric‐field treatment was also reported to induce the accumulation of mobile ions at opposite metal electrodes by polarization‐induced ion migration.^[^ ^61^ ^]^ Here, it is believed that the redistribution of ionic defects, including MA^+^ and X^−^, breaks the macroscopic symmetry of perovskite structure, thus leading to an unexpectedly high electromechanical response despite being considered cubic centrosymmetric structure. Accordingly, the electric field induces the separation of oppositely charged ionic defects and thus leads to extra space‐charge polarization. Figure 4c presents the dependence of the harvesting performance on the load resistance of the halide films, which show the voltage increasing with load resistance, as anticipated. The maximum output voltage was ≈23.5 V at 10^7^ Ω for the strained MAPbI_3_ film, with a peak output power of ≈182 µW. These values are the highest among values reported thus far for halide thin‐film‐based harvesters.

## 3 Conclusion

High‐quality flexible thin films of MAPbI_3_, MAPbBr_3_, and MAPbCl_3_ were prepared by two‐step solution deposition on a plastic substrate for bending‐driven piezoelectric energy harvesting. A strain engineering approach was applied to the thin films to induce a tensile strain parallel to the film thickness to allow lattice extension in the out‐of‐plane direction. The ferroelectricity of MAPbI_3_ thin films was proven by the hysteresis loop and structural analysis, with quantitative estimations of the distortion of coupled PbI_6_ octahedra and the atomic displacement within each octahedron. The piezoelectricity of cubic MAPbBr_3_ and MAPbCl_3_ was assumed to originate from soft polarity modes and relatively low elastic moduli, which originated from hydrogen bonds between MA^+^ and X^−^ ions and local asymmetric structure, with contributions by vacancies to the space‐charge polarization. The grain boundary potential and the estimated bond strength and tolerance factor support well the experimental trends of piezoelectricity depending on the anions. The optimized peak voltage/current values after adjusting the in situ lattice strain and electric bias were 23.1 V/1703 nA for MAPbI_3_, 5.6 V/176 nA for MAPbBr_3_, and 3.3 V/141 nA for MAPbCl_3_. The output values achieved by MAPbI_3_ are the highest recorded values among those of reported perovskite halide thin‐film harvesters.

## 4 Experimental Section

### Preparation of In Situ Strained Thin Films

Flexible MAPbX_3_ (X = I, Br, Cl) thin films were prepared on a 125‐µm‐thick ITO/PEN substrate via a two‐step sequential deposition process using two precursor solutions, PbX_2_ and MAX. To deposit the MAPbI_3_ thin films, the PbI_2_ precursor was prepared by dissolving lead iodide (PbI_2_, 99.99%, TCI, Japan) in *N*,*N*‐dimethylformamide (DMF, anhydrous, 99.8%, Sigma‐Aldrich, USA) at a concentration of 1.5 m. Identical solvents and concentrations were used, except for the raw materials of lead bromide (PbBr_2_, 99.99%, TCI, Japan) and lead chloride (PbCl_2_, 99.99%, TCI, Japan) to prepare the PbX_2_ precursors. The three MAX precursors (Greatcell Solar Materials, Australia) were separately prepared by dissolving methylammonium iodide (MAI, 99.9%), methylammonium bromide (MABr, 99.9%), and methylammonium chloride (MACl, 99.9%) in anhydrous isopropyl alcohol (IPA; C_3_H_8_O, anhydrous, Daejung, Korea) at concentrations of 20, 14.6, and 8.6 mg mL^−1^, respectively.

Prior to depositing the precursor solution, the 4 cm × 4 cm ITO/PEN substrate was surface treated in O_2_ (20 sccm) plasma at 100 W for 5 min after ultrasonication in an IPA bath for 10 min. An aqueous solution of PEDOT:PSS (AI4083, Heraeus, Germany) was then spin coated onto the plasma‐treated ITO/PEN at 3500 rpm for 30 s and dried at 75 °C for 20 min. An additional PEDOT:PSS interlayer was selected to facilitate the formation of a loosely packed first layer that allowed the easy penetration of the second precursor, as previously reported.^[^ ^5^, ^31^ ^]^ The PbX_2_ solution was first spin coated onto the PEDOT:PSS/ITO/PEN at 4000 rpm for 30 s and annealed at 75 °C for 15 min. The annealed PbX_2_ sample was then bent in a convex or concave manner while controlling the curvature of the bent sample using a position‐adjustable fixture. The bent sample was then dip coated in the MAX solution at 50 °C for 15 min, rinsed with IPA, and dried at 50 °C for 10 min. The bent state recovered to a flat state by releasing the deposited film from the fixture after drying. Different in situ strain *ε_i_* values of −0.73%, −0.32%, 0%, +0.32%, and +0.73% were selected for the deposition of MAPbI_3_ thin films, in which the negative (positive) sign indicates compressive (tensile) strain. Only the optimal compressive strain of −0.73% was applied to the MAPbBr_3_ and MAPbCl_3_ thin films. The procedure for calculating in situ strain *ε_i_* is available in the Supporting Information with the schematic of neutral position in the bent structure in Figure S14, Supporting Information.

### Preparation of Piezoelectric Energy Harvesters

Piezoelectric energy harvesters were fabricated by covering the in situ deposited MAPbX_3_ films with another PDMS‐coated ITO/PEN substrate. A PDMS (Sylgard 184, Dow Corning, USA) solution consisting of the base monomer with 10 wt% curing agent was spin coated onto a 50‐µm‐thick ITO/PEN substrate at 7000 rpm for 60 s and pre‐cured at 100 °C for 3 min. The pre‐cured PDMS layer was attached to the MAPbX_3_ film in the flat state and then cured again at 70 °C for a longer duration of 1 h to eliminate the potential gap between the PDMS and halide in the completed harvester structure of PEN/ITO/PDMS/MAPbX_3_/PEDOT:PSS/ITO/PEN with an effective area of 14 cm^2^, as reported elsewhere.^[^ ^13^, ^62^ ^]^ Cu wires were externally connected to both sides of the exposed ITO before passivating the harvester with PI tape.

### Measurement and Characterization

The crystal structure of the in situ strained MAPbX_3_ films was examined using an X‐ray diffractometer (SmartLab, Rigaku, Japan) operated in scan step mode with a step size of 0.01° and a dwell time of 3 s per step in the scan range of 10–50° under an incident glancing angle of 0.5°. Each peak position was determined using a Gaussian fitting. Structural information, including the interatomic distance and bond angle, was estimated by VESTA software using the lattice parameters extracted from the HR‐XRD patterns. Cross‐sectional images were obtained using a field‐emission scanning electron microscope (FE‐SEM; JSM‐7610F, JEOL, Japan). The film surface was further characterized to confirm the existence of lattice strain using high‐resolution XPS; (VG ESCALAB 220i‐XL, Thermo Fisher Scientific, USA) with Al‐K*α* photons (1486.6 eV) at a base pressure of 1.1 × 10^−10^ Torr in an ultrahigh vacuum chamber. The XPS spectra were calibrated using the C 1s peak. The PL spectra were obtained using Raman spectroscopy (inVia RE04, Renishaw, UK) with an excitation wavelength of 532 nm. The 50‐nm‐thick Au top electrode with an active area of ≈0.0314 mm^2^ was deposited for dielectric and polarization measurements. Dielectric constant *ε_r_* was obtained as a function of frequency ranging from 7 × 10^2^ to 10^5^ Hz using an impedance analyzer (HP 4194A, Hewlett‐Packard, USA). The P–E loops and current–electric field curves were obtained at a frequency of 10 Hz by using the dynamically hysteresis method (DHM) based on triangular wave pulses. The n/p‐type determination and Hall mobility measurements were carried out using a Hall effect measurement system (HMS‐2000, Ecopia, USA) and the four‐point probe method.

The piezoelectric properties of the halide films were characterized by PFM (Nanoscope V multimode, Bruker, USA) using a conductive Pt/Ir‐coated Si cantilever tip. The PR amplitude was measured by scanning the film surface (3 µm × 3 µm) at a driving amplitude of 4 V in lock‐in mode under a variable AC voltage *V*_ac_. The effective out‐of‐plane piezoelectric coefficient *d*_33,eff_ was estimated from the peak amplitude using the relation *A*_deflection_ = *d*_33,eff_ × *V*_ac_ / 16 where *A*_deflection_ is the peak amplitude. The surface potential was characterized using amplitude‐modulated KPFM. The bias frequency was set to the second resonance frequency of the cantilever at ≈315.4 kHz under an AC bias of 2 V. P–E hysteresis loops were investigated in a ferroelectric module (TF analyzer 2000, aixACCT, Germany) in dynamic hysteresis measurement mode.

The harvesting performance was assessed under periodic bending in the frequency range of 0.3–3.4 Hz and at bending strains of 0.13–0.47% using a one‐axis high‐speed fatigue machine (CTLM500, Ceratorq Inc., Korea). The output voltage was measured using a nanovoltmeter (Keithley 2182A, ValueTronics, USA) operating at an internal resistance of 10 MΩ, and the output current was determined with a galvanostat system (IviumStat, Ivium Technologies, Netherlands) operating at an internal resistance of 1 MΩ. To calculate the power for the optimal harvesters, the output voltage was measured by changing the load resistance *R*_L_ in the range of 10^2^ to 10^7^ Ω using a high‐speed peak detector (V64, Ceratorq Inc., Korea).

## Conflict of Interest

The authors declare no conflict of interest.

## Supporting information

Supporting information

## Data Availability

The data that support the findings of this study are available from the corresponding author upon reasonable request.
