# Supplementary material for: Anion‐Dependent Polarization and Piezoelectric Power Generation in Hybrid Halide MAPbX3 (X = I, Br, and Cl) Thin Films with Out‐of‐Plane Structural Adjustments
Source: Adv Sci (Weinh). 2022 Dec 1;10(4):2204462. doi: 10.1002/advs.202204462 (PMC9896056; doi:10.1002/advs.202204462)
Supplement: Supplementary file 1 — Supporting information [file ADVS-10-2204462-s001.pdf]

ADVANCED SCIENCE
Open Access

Supporting Information

for *Adv. Sci.*, DOI 10.1002/advs.202204462

Anion-Dependent Polarization and Piezoelectric Power Generation in Hybrid Halide $MAPbX_3$ (X = I, Br, and Cl) Thin Films with Out-of-Plane Structural Adjustments

*Da Bin Kim, Kyeong Su Jo, Kwan Sik Park and Yong Soo Cho**

Supporting Information

Anion-Dependent Polarization and Piezoelectric Power Generation in Hybrid Halide $MAPbX_3$ (X = I, Br, and Cl) Thin Films with Out-of-Plane Structural Adjustments

*Da Bin Kim[#], Kyeong Su Jo[#], Kwan Sik Park, and Yong Soo Cho**

D. B. Kim, K. S. Jo, K. S. Park, Prof. Y. S. Cho
Department of Materials Science and Engineering, Yonsei University, Seoul 03722, Republic of Korea
E-mail: ycho@yonsei.ac.kr

D. B. Kim
Department of Electrical and Computer Engineering, University of Toronto, Toronto, Ontario M5S 3G4, Canada

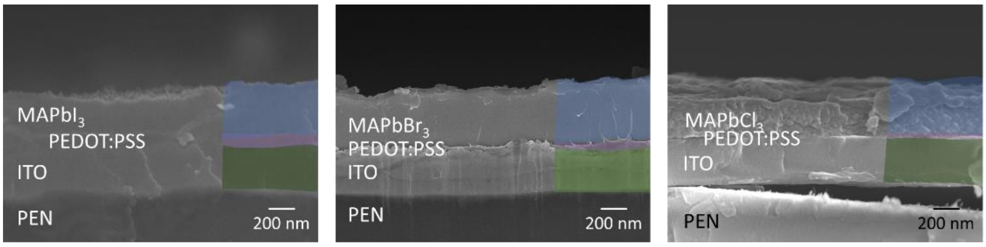

Figure S1. Cross-sectional SEM images of $MAPbI_3$, $MAPbBr_3$, and $MAPbCl_3$ thin films processed by two-step solution deposition.

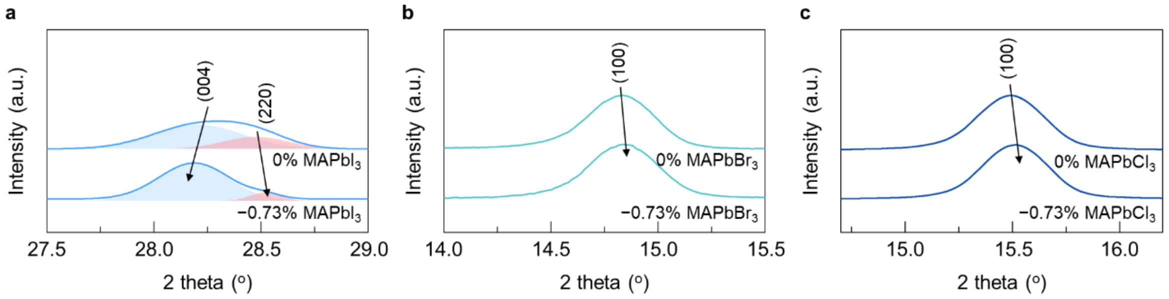

Figure S2. Highlighted HR-XRD peaks of (a) the (004) and (220) planes in $MAPbI_3$, (b) the (100) plane in $MAPbBr_3$, and (c) the (100) plane in $MAPbCl_3$, indicating peak shifts with the introduction of in situ strain ε_i.

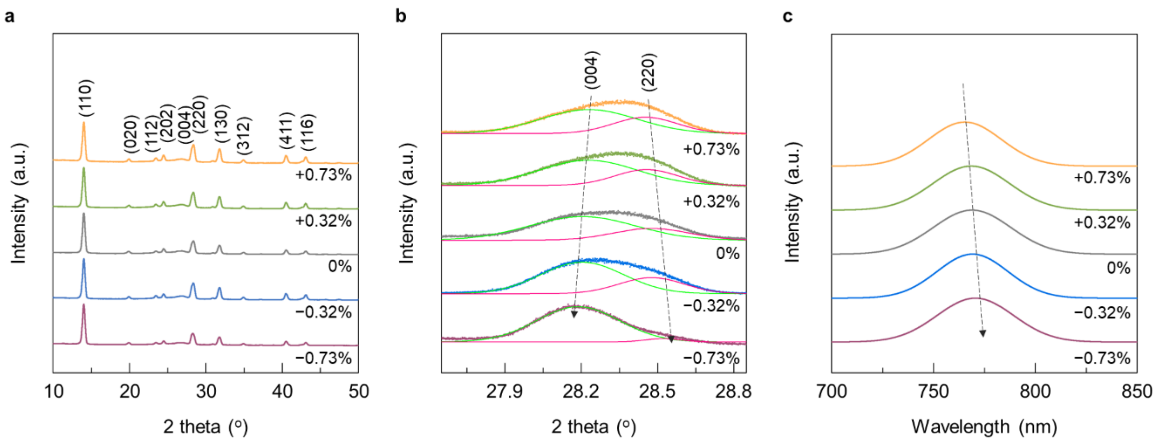

Figure S3. (a) Full-scale HR-XRD patterns of the $MAPbI_3$ thin films processed with more in situ strain ε_i values from +0.73% (tensile) to −0.73% (compressive). (b) XRD peaks of the (004) and (220) planes in the ε_i range of +0.73% to −0.73%. (c) PL spectra of the $MAPbI_3$ thin films processed with ε_i ranging from +0.73% to −0.73%.

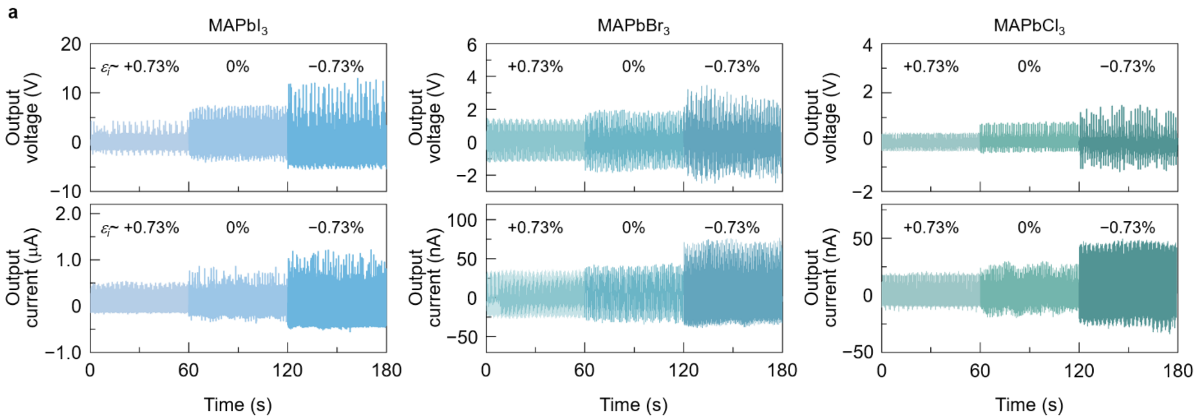

Figure S4. Output voltage and output current generated for the +0.73%-strained, unstrained and −0.73%-strained $MAPbI_3$, $MAPbBr_3$, and $MAPbCl_3$ thin films, which were measured at a bending strain of 0.47% and a frequency of 2.9 Hz.

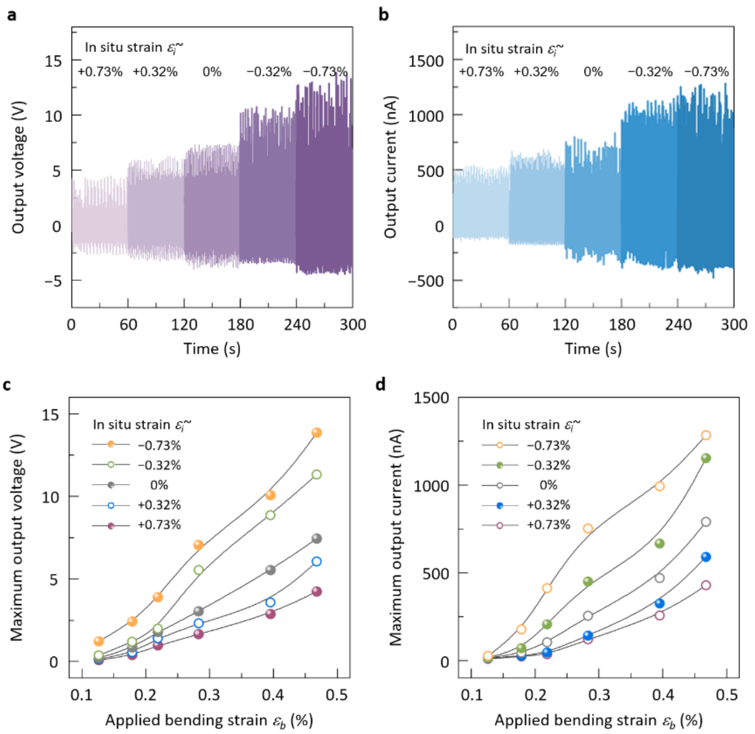

Figure S5. (a) Output voltage and (b) output current of the $MAPbI_3$ thin-film harvesters processed with different in situ strain values ε_i under the optimal bending conditions of a bending strain and frequency of 0.47% and 2.9 Hz, respectively. Variations in the (c) maximum voltage and (d) maximum current with the increasing bending strain for the energy harvesters processed with ε_i of −0.73%, −0.32%, 0%, +0.32%, and +0.73%.

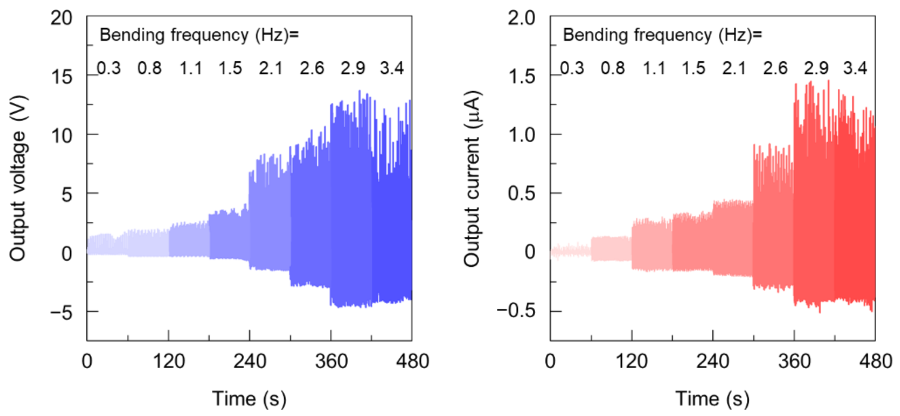

Figure S6. Piezoelectric energy harvesting performance: output voltage (left) and current (right) with the increasing bending frequency from 0.3 to 3.4 Hz at a bending strain of 0.47% for the −0.73%-strained $MAPbI_3$ thin-film harvester.

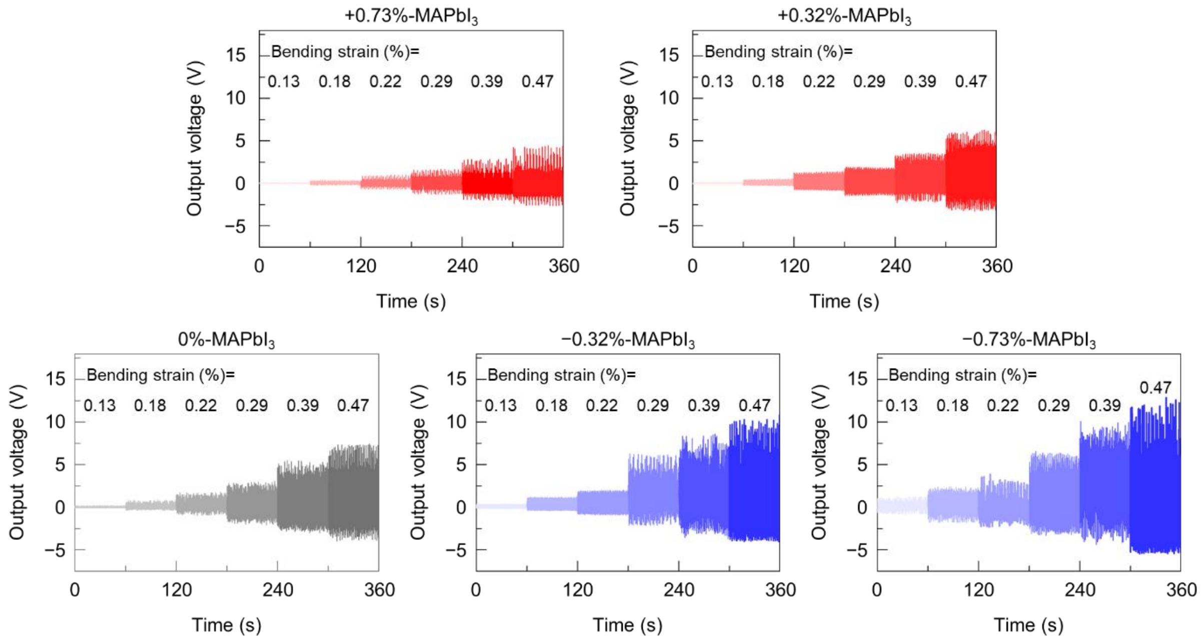

Figure S7. Piezoelectric output voltage with the increasing bending strain from 0.13% to 0.47% for the $MAPbI_3$ thin-film harvesters processed with in situ strain ε_i ranging from +0.73% to −0.73% at a bending frequency of 2.9 Hz.

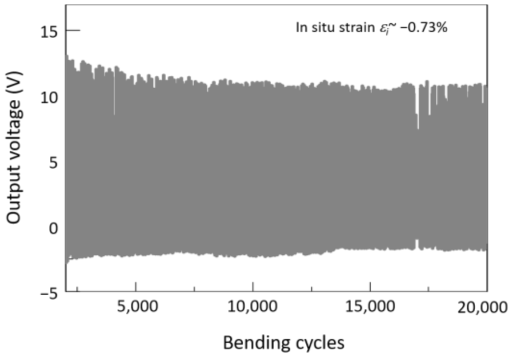

Figure S8. Long-term output voltage generated by the −0.73%-strained $MAPbI_3$ harvester over 20,000 bending cycles at a bending strain and frequency of 0.47% and 2.9 Hz, respectively.

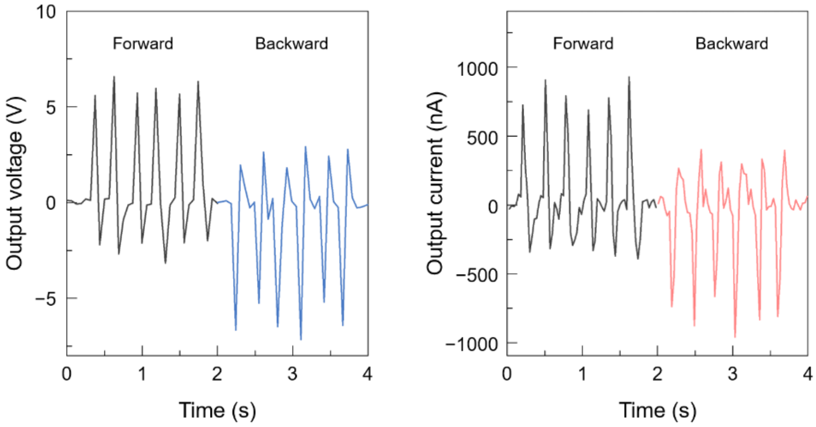

Figure S9. Polarity switching behavior with forward and backward connections, suggesting that the output voltage and current are generated by the piezoelectric halide layer of $MAPbI_3$.

Table S1. Reported d_{33} in organic-inorganic cubic halides, which were obtained by experiments.

Materials	Structure	Type	d_{33}	Measurement	Reference
$FASnI_3$	Cubic (*Pm-3m*)	Thin film (800 nm)	38 pm/V	PFM	*ACS Energy Lett.* **2019**, *4*, 1004-1011
$FAPbBr_3$	Cubic (*Pm-3m*)	Thin film (500 nm)	25 pm/V	PFM	*Adv. Funct. Mater.* **2016**, *26*, 7708–7716
$MASnBr_3$	Cubic (*Pm-3m*)	Thin film (N/A)	2.7 pm/V	PFM	*ACS Appl. Mater. Interfaces* **2020**, *12*, 16469−16480
$MASnI_3$	Cubic (*Pm-3m*)	Thick film (5.1 µm)	20.8 pm/V	PFM	*Nano Energy* **2019**, *57*, 911-923

Here, FA (Formamidinium): $(NH_2)_2CH$ and MA: CH_3NH_3

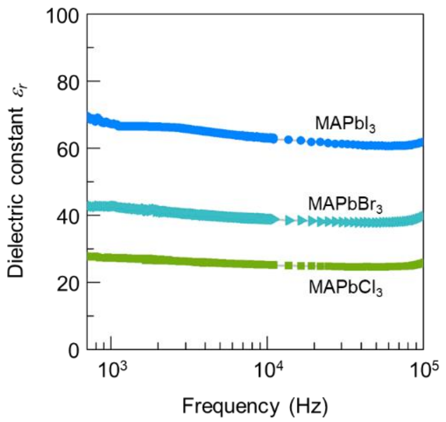

Figure S10. Dielectric constant versus frequency in $MAPbX_3$ (X=I, Br, and Cl) thin films.

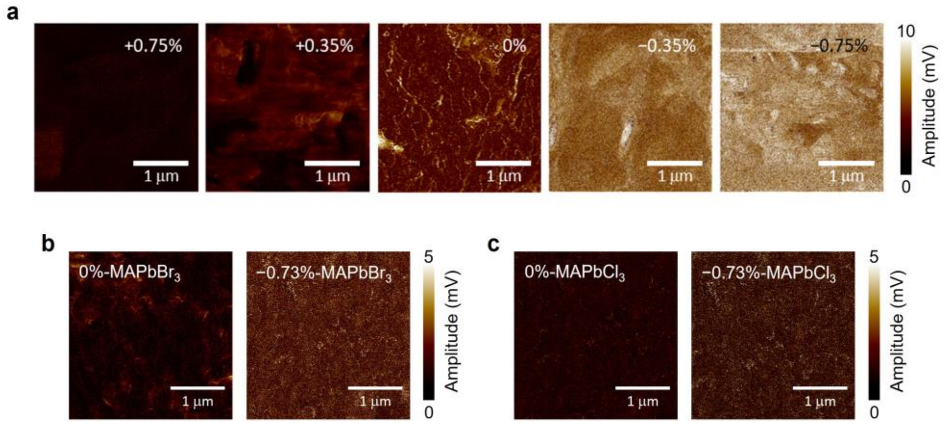

Figure S11. PR amplitude images of the unstrained and strained (a) $MAPbI_3$, (b) $MAPbBr_3$, and (c) $MAPbCl_3$ thin films.

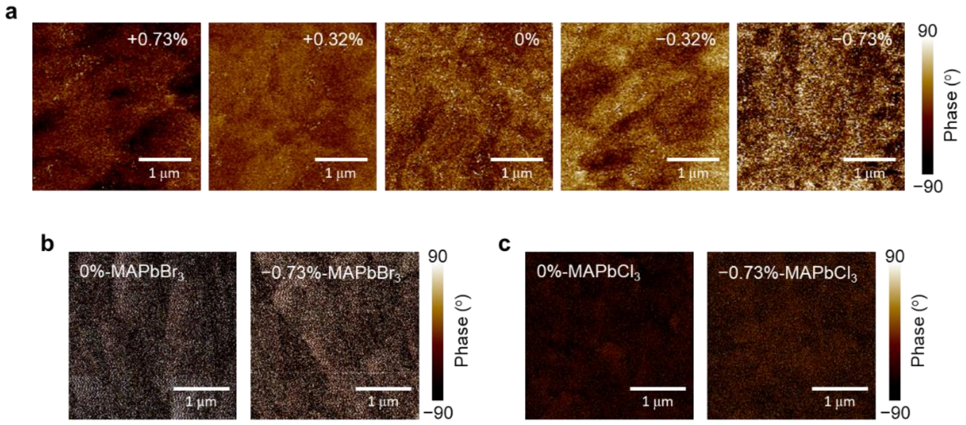

Figure S12. PR phase images of the unstrained and strained (a) $MAPbI_3$, (b) $MAPbBr_3$, and (c) $MAPbCl_3$ thin films.

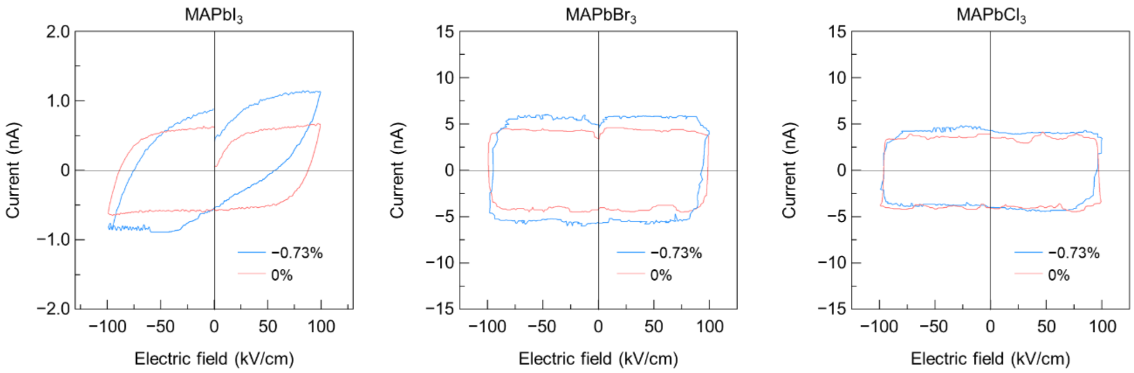

Figure S13. Current–electric field curves of the unstrained and −0.73%-strained $MAPbI_3$, $MAPbBr_3$, and $MAPbCl_3$ thin films, demonstrating apparent ferroelectric behavior only in $MAPbI_3$.

Table S2. Structural information of the strained $MAPbI_3$ thin films extracted from the HR-XRD results. A negative (positive) sign indicates compressive (tensile) strain.

In situ strain ε_i (%)	Lattice constants (Å)		Volume V (Å^3)
	a	c	
+0.73	8.2873	12.6347	993.11
+0.32	8.2824	12.6357	992.88
0	8.2741	12.6459	992.42
−0.32	8.2638	12.6462	992.36
−0.73	8.2566	12.6535	989.86

Table S3. Estimated variations in the crystal structural attributes of the perovskite $MAPbI_3$ structure for various values of in situ strain ε_i ranging from +0.73% to –0.73%. Here, σ^2_{oct} is the bond angle variance, and D is the distortion index.

In situ strain ε_i (%)	+0.73	+0.32	0	−0.32	−0.73
Average Pb–I bond length (Å)	3.2452	3.2449	3.2444	3.2443	3.2415
PbI_6 octahedron volume (Å^3)	45.334	45.323	45.302	45.300	45.185
I_I–Pb–I_{II} bond angle (°)	95.1421	95.1704	95.1714	95.1906	95.1937
I_{II}–Pb–I_{VI} bond angle (°)	84.8579	84.8296	84.8286	84.8094	84.8063
I_V–Pb–I_{VI} bond angle (°)	89.8842	89.8829	89.8829	89.882	89.8818
D	0.0123	0.0128	0.0128	0.0131	0.0132
σ^2_{oct} (deg.^{2})	11.3654	11.4505	11.4533	11.5113	11.5209

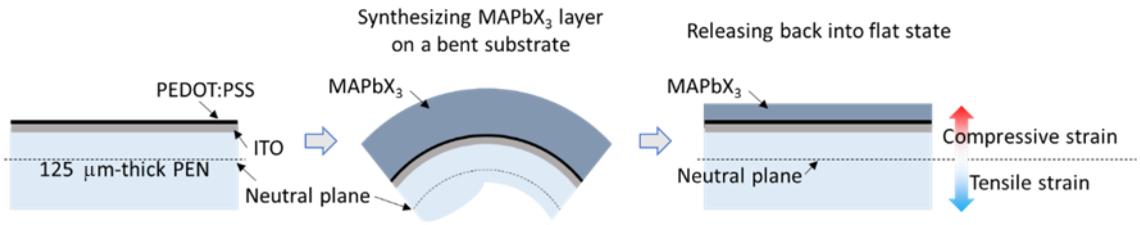

Figure S14. Schematic illustration of the $MAPbX_3$/PEDOT:PSS/ITO/PEN structure with the projected position of neutral plane in the bent state. Refer to the description below on the detailed procedure for the calculation of in situ strain ε_i.

NOTE: Two-step procedure for the calculation of in situ strain ε_i

1) *The estimation of the neutral plane*

The location of the neutral plane, *y*, underneath from the top surface was calculated with considering the contribution of each layer, using the following equation reported for the multi-layered composite structure (*Science* **2009**, *325*, 977-981):

$$y = \frac{\sum_{i=1}^{4}(\sum_{j=1}^{i} t_j - \frac{t_i}{2})}{\sum_{i=1}^{4} E_i^* t_i}$$

where $E^*_i = E_i/(1-\nu_i^2)$ (here, E_i and ν_i, are the Young's modulus and the Poisson's ratio of the *ith* layer, respectively), t_i is thickness of *ith* layer, and t_j is the thickness of *jth* layer.

The following data were used for the calculation:

E_1=15.9 GPa, ν_4= 0.317 for 486-nm-thick $MAPbI_3$ thin film (*Adv. Funct. Mater.* **2021**, *31*, 2007131)

(or E_1=17.8 GPa, ν_1= 0.319 for 464-nm-thick $MAPbBr_3$ thin film (*J. Mater. Chem. A* **2015**, *3*, 18450)

(or E_1=19.9 GPa, ν_1= 0.330 for 450-nm-thick $MAPbCl_3$ thin film (*J. Mater. Chem. A* **2015**, *3*, 18450)

E_2=2.4 GPa, ν_2=0.5 for 70-nm-thick PEODT:PSS layer (*Key Eng. Mater.* **2007**, *345*, 1189)

E_3=100 GPa, ν_3=0.2 for 350-nm-thick ITO electrode (*Thin Solid Films* **2004**, *460*, 156)

E_4=6.1 GPa, ν_4=0.33 for 125-μm-thick PEN substrate (*J. Soc. Inf. Disp.* **2007**, *15*, 1075)

The resulting neutral plane *y* was 60.25 μm for $MAPbI_3$, 60.19 μm for $MAPbBr_3$, and 60.12 μm for $MAPbCl_3$.

2) *The calculation of in situ strain in the halide layer*

The strain value in the halide layer was calculated by the relation of $\varepsilon_i = y/r$, where *r* is the radius of bending curvature. The *r* value was calculated by the following equation.

$$r = \frac{L_0}{2\pi\sqrt{\frac{\Delta L}{L_0} - \frac{\pi^2 t^2}{12 L_0^2}}}$$

where L_0 is the length of PEN substrate. The value of ΔL corresponds to the reduced length in parallel to the flat state after bending the substrate. For example, if the original length (L_0) of 40 mm was shortened to the length (ΔL) of 24 mm (corresponding to the bending curvature r of 8.2188 mm), the final ε_i values were obtained as being 0.7331% for $MAPbI_3$, 0.7323% for $MAPbBr_3$, and 0.7315% for $MAPbCl_3$. The strain values are listed in the bottom of a Table below, which were used as the maximum values for this study.

Selected calculated values of in situ strain ε_i for $MAPbX_3$ (X=I, Br, and Cl) thin films

L_0 (mm)	ΔL (mm)	L_0-ΔL (mm)	r (mm)	ε_i (%)		
				$MAPbI_3$	$MAPbBr_3$	$MAPbCl_3$
40	2	38	28.4728	0.2116	0.2114	0.2111
40	4	36	20.1325	0.2993	0.2990	0.2986
40	6	34	16.4379	0.3665	0.3662	0.3657
40	8	32	14.2355	0.4232	0.4228	0.4223
40	10	30	12.7326	0.4732	0.4727	0.4722
40	12	28	11.6232	0.5184	0.5178	0.5172
40	14	26	10.7610	0.5599	0.5593	0.5587
40	16	24	10.0659	0.5986	0.5980	0.5973
40	18	22	9.4903	0.6349	0.6342	0.6335
40	20	20	9.0032	0.6692	0.6685	0.6677
40	22	18	8.5842	0.7019	0.7012	0.7003
40	24	16	8.2188	0.7331	0.7323	0.7315
